# Preoperative serum C- reactive protein: a prognostic marker in patients with upper urinary tract urothelial carcinoma

**DOI:** 10.1186/1471-2407-13-101

**Published:** 2013-03-06

**Authors:** Barbara Stein, Andres Jan Schrader, Gerd Wegener, Christoph Seidel, Markus A Kuczyk, Sandra Steffens

**Affiliations:** 1 Department of Urology, Hannover Medical School, Carl-Neuberg-Str. 1, Hannover, D-30625, Germany; 2 Department of Urology, Ulm Medical School, Ulm, Germany; 3 Cancer Center, Hannover Medical School, Hannover, Germany; 4 Department of Oncology, Hannover Medical School, Hannover, Germany

**Keywords:** UUT-UC, Biomarker, C-reactive protein, Aggressivness, Prognosis, Survival

## Abstract

**Background:**

To analyse the prognostic significance of preoperative C-reactive protein (CRP) serum level in patients with upper urinary tract urothelial carcinoma (UUT-UC).

**Methods:**

We evaluated 158 UUT-UC patients who had undergone surgery in the University Hospital of Hannover (MHH). 143 (89.4%) suffered from cancer in the renal pelvis, 13 (8.1%) patients presented with tumour located in the ureter. A preoperative CRP value was available for 115 patients. The mean (median) follow-up for these patients was 28.3 (15.1) months.

**Results:**

The median (mean) CRP value of all evaluable patients was 10.0 (40.7) mg/l. The CRP-level, stratified into two subgroups (CRP ≤5 vs. >5 mg/l), correlated significantly with muscle invasive tumour stage (36.4 vs. 78.9%; p<0.001), the risk of presenting nodal disease (4.5 vs. 26.8%; p=0.002) and distant metastasis (2.3 vs. 16.9%; p<0.016). The Kaplan-Meier 5-year cancer specific survival (CSS) rates were 54.2 and 26.4% for patients with preoperative CRP levels ≤ and >5 mg/l, respectively (p<0.006). Next to age and the presence of metastasis, multivariate analysis also identified CRP as a continuous variable as an independent prognosticator for CSS.

**Conclusions:**

A high preoperative serum CRP level is associated with locally advanced and metastatic disease in patients with UUT-UC. Its routine use could allow better risk stratification and risk-adjusted follow-up of UUT-UC patients.

## Background

Upper urinary tract urothelial carcinoma (UUT-UC) accounts for 5–7% of all urothelial malignancies in adults; and its incidence increased steadily over the last 20 years [1]. Thus UUT-UC, compared to bladder cancer, is still relatively uncommon; however, it is often a highly aggressive tumour and the prognosis, in general, is poorer than that for urothelial cancer of the bladder [1].

As patients' clinical courses vary and are difficult to predict, the stratification of patients to appropriate postoperative surveillance programs and different therapeutic strategies tailored to the individual risk of cancer progression is helpful. Tumour stage, pathological grade, tumour location, lymph node involvement, lymphovascular invasion and surgical procedure are known prognostic factors [2-6]. However, all of these are postoperative factors, therefore identifying preoperative prognostic factors, including a serum biomarker, would allow a better therapeutic approach. Particularly biomarkers in body fluids could offer the opportunity for more objective and reproducible measurement and risk stratification prior to surgery.

C-reactive Protein (CRP) is an acute phase protein produced almost exclusively by the liver. CRP plasma levels can increase up to 1000-fold in response to microbial infection, trauma, infarction, autoimmune, or malignant diseases. Elevated CRP levels can be a result of an underlying cancer and a premalignant state, respectively, as well as due to tumour growth associated tissue inflammation. A study published in 2009 by Allin et al. [7] involving 10,408 individuals showed that elevated CRP is associated with increased risk of cancer, e.g. lung or colorectal malignancies. Furthermore, an elevated CRP level was associated with an early death, even in patients without metastases [7].

McArdle et al. [8] were able to show that CRP, next to prostate specific antigen (PSA), could serve as an additional independent prognostic marker for tumour-specific survival in metastatic castration-resistant prostate cancer. Furthermore, several studies published in recent years including from 40 up to 1,161 patients indicated that the preoperative CRP level could also be associated with RCC-specific mortality [9-17].

Concerning urothelial carcinoma, Trichopoulos et al. [18] revealed that elevated CRP can be related to a higher risk of developing bladder cancer. In patients with advanced bladder cancer undergoing chemotherapy elevated CRP levels were shown to be associated with a poor clinical outcome [19]. To our knowledge only one study published so far was able to show that in patients undergoing surgery for UUT-UC an increased CRP level seems to be asscociated with poor survival [20]. Therefore, in this study including 115 patients, we validated the potential pre-operative prognostic significance of CRP in patients undergoing surgery for UUT-UC.

## Methods

### Patients and tumour characteristics

This study included 158 patients with complete patient and tumour specific characteristics who underwent surgery form 1981–2011 for UUT-UC at the Hannover University Medical Centre (MHH). None of the patients had received preoperative chemotherapy. The regional lymph nodes were dissected in patients with enlarged nodes during surgery or in case of pathological findings on the pre-operative CT scan; an extended lymphadenectomy was not used routinely. 43 patients were excluded because their preoperative CRP levels were unavailable. The ethical committee of the MHH approved the study. The histological tumour subtype was determined according to the 1997 UICC classification. Staging was based on the 2002 TNM classification. Information on patients´ and tumour characteristics, such as age, sex, stage, presence of regional lymph node or distant metastases, histological subtype, tumour grade according to the Who classification, and CRP-value, was obtained from our computerized institutional databases. The pre-operative CRP-value was categorized into two groups CRP≤5 mg/l and >5 mg/l according to the suggestion of Saito et al. [20].

### Follow up

After surgery patients underwent urinary cytology and cystoscopy every 3 months for the first 2 years. In addition, CT and/or MRI were used every 6 months for 5 years and annually thereafter. The duration of the follow-up was calculated from date of surgery to the date of death or last follow-up. Death was assessed as either cancer-related or -unrelated. The primary end point of this study was cancer-specific survival (CSS). Information about the exact date as well as cause of death for each patient was received from the patient’s general practitioner, a close family member or the patient’s hospital records if she/he had been followed up or died in our institution. Follow-up assessment ended in October 2011. Until then, all patients’ data were updated at least every 12 months on a regular basis.

### Statistical methods

Continuous variables were reported as mean value and standard deviation (SD) or median value and interquartile ranges (IQR) in the case of parametric or non-parametric distribution, respectively. Chi^2^ and Fisher’s exact tests were conducted to assess correlations of nominal covariate distributions and CRP-groups. The t-test and ANOVA (in case of parametric) or the Mann–Whitney-U-test (in case of non-parametric distribution) was applied to compare metric variables between two or more subgroups.

Receiver operation characteristics (ROC) curves were constructed to assess the potential of preoperative CRP to predict muscle invasive disease, poor differentiation, and the presence of metastasis at the time of surgery.

Kaplan-Meier estimates of survival time were calculated, and subgroups were compared by the log rank test statistic. Multivariate Cox regression models were used to assess the association between survival and CRP-levels adjusted for different clinical and patient covariates (i.e., age, sex, tumour stage and grade, and metastatic status). SPSS 19.0 was used for statistical assessment. In all tests, a two-sided p<0.05 was considered to indicate significance.

## Results

Our patient population with an available pre-surgical CRP value consisted of 83 (72.2%) men and 32 (27.8%) women had a mean (median) age of 65.6 (66.8) years (34–90). The median body mass index (BMI) for all patients was 25.0 kg/m^2^ (IQR, 22.6 – 28.1). 107 (93.9%) suffered from cancer in the renal pelvis, 7 (6.1%) patients presented with tumour located in the ureter. Detailed patients’ and tumour characteristics including stage and grade are summarized in Table 1.

**Table 1:** Association between different patient and cancer-specific variables with the pre-operative CRP value

**Variable**	**CRP ≤5 mg/l, n (%)**	**CRP >5 mg/l, n (%)**	**p-value**	**Test**
Age, mean [years] ±SD	62.8 (±12.2)	67.3 (±11.8)	0.054	t-test
BMI, mean [kg/m^2^] ±SD	25.7 (±3.8)	25.4 (±4.2)	0.67	t-test
Sex			0.67	Fisher
female	11 (25.0)	21 (29.6)		
male	33 (75.0)	50 (70.4)		
Location			0.71	Fisher
renal pelvis	42 (95.5)	65 (92.9)		
ureter	2 (4.5)	5 (7.1)		
Stage				Fisher
superficial (pT<2)	28 (63.6)	15 (21.1)	<0.001	
invasive (≥pT2)	16 (36.4)	56 (78.9)		
Metastasis ^1^			0.001	Fisher
N / M -	41 (93.2)	48 (67.6)		
N / M +	3 (6.8)	23 (32.4)		
Grade			<0.001	chi-square
G1	12 (27.3)	7 (9.9)		
G2	24 (54.5)	18 (25.4)		
G3	8 (18.2)	46 (64.8)		

^1^ at time of surgery.

The median / mean follow-up was 15.1 / 28.3 months (IQR, 7.2 – 37.7). By the last day of data acquisition, 39 (33.9%) patients had died from their tumour disease.

### Clinical parameters

The mean (median) pre-operative CRP value of all 115 evaluable patients was 40.7 (10.0) mg/l. The mean (median) CRP value in the two subgroups (CRP≤5, n=44 and >5 mg/l, n=71) was 2.3 (2.0) and 64.6 (45.0) mg/l, respectively. Both groups were comparable concerning the distribution of sexes (p=0.67; Fisher’s exact test, Table 1), the BMI value (p=0.67; t-test) and tumour location (p=0.71, Fisher’s exact test; Table 1) as well as age, even though patients with a CRP >5 mg/l tended to be older (p=0.054; t-test).

### Tumour-specific parameters

The CRP-level correlated significantly with the tumour stage: 36.4 and 78.9% of all patients with a CRP ≤ and >5 mg/l suffered from muscle invasive disease (pT≥2) at the time diagnosis (p<0.001, Fisher’s exact test). Accordingly, the median CRP value was significantly lower in those patients with superficial compared to invasive cancer (4.0 vs. 19.5 mg/l; p<0.001, Mann–Whitney-U test). The risk of presenting nodal disease (p=0.002, Fisher’s exact test) or distant metastasis (p=0.016, Fisher’s exact test; Table 1) also increased significantly in patients with a CRP value >5 mg/l. Moreover, elevated CRP-levels were found in 9.9, 25.4 and 64.8% of patients with G1, G2 and G3 tumours, respectively (p<0.001, Chi^2^ test).

### Receiver-operating characteristic (ROC) analysis

Applying ROC analyses, the CRP value as a continuous metric variable exhibited an AUC (95% CI) of 0.73 (0.64 – 0.83; p<0.001) for the presence of metastatic disease at the time of surgery (lymph node or visceral metastasis), of 0.76 (0.67 – 0.85; p<0.001) for poor tumour differentiation (G3), and of 0.70 (0.60 – 0.80; p<0.001) for the presence of a muscle invasive stage.

### Clinical course and oncological outcome

**Figure 1:**
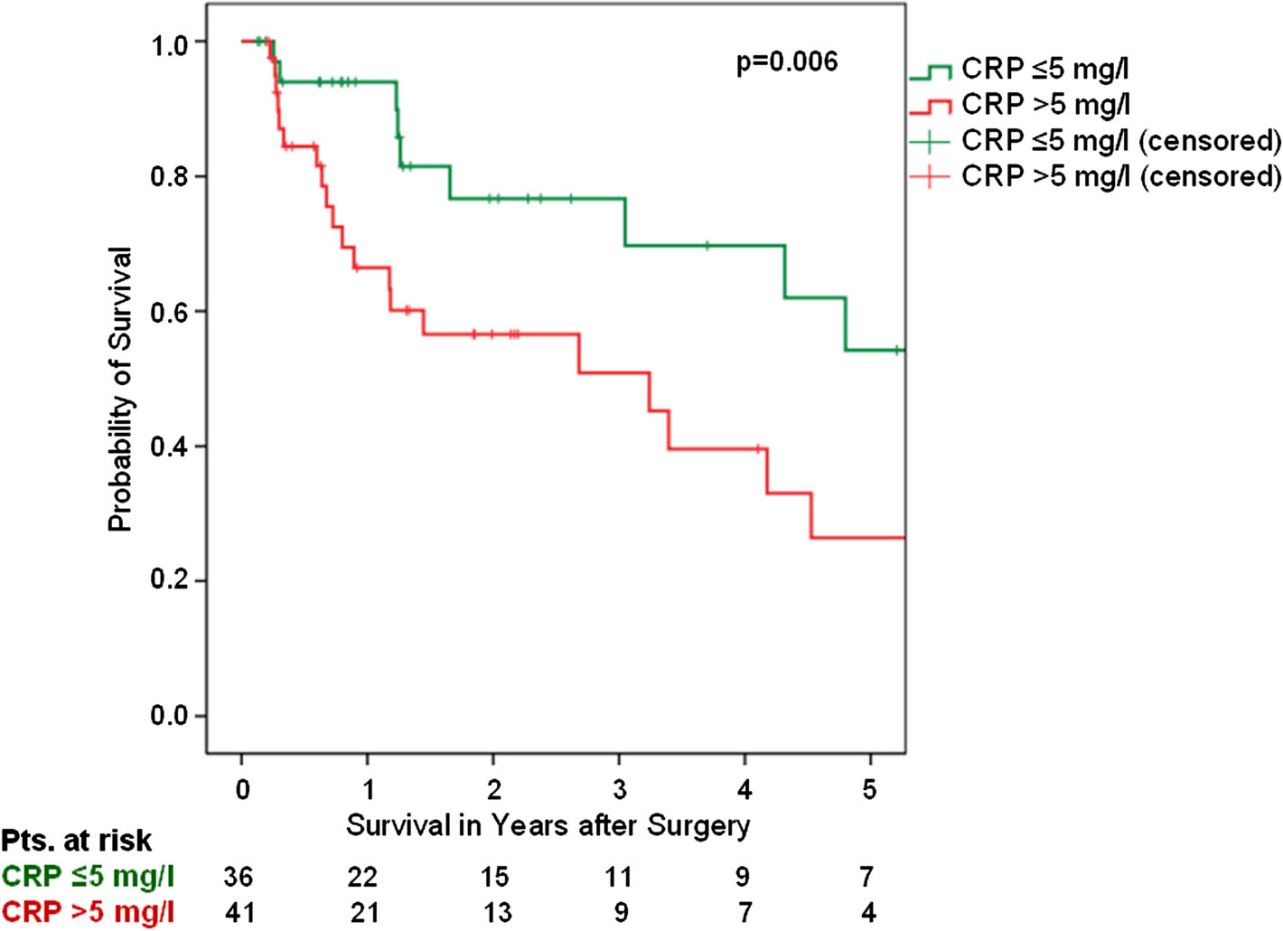
**Cancer-specific survival (Kaplan-Meier) for all UUT-UC patients plotted against the pre-operative CRP-group.** The 5-year survival rates were 54.2 and 26.4%, respectively, for all evaluable patients (n=77) with a CRP of ≤5 mg/l (n=36) and >5 mg/l (n=41), respectively (p=0.006, log rank).

The median survival for all evaluable patients was 50.1 months (Kaplan-Meier). Applying univariate Cox-Regression calculation, an elevated BMI (p=0.75) or male sex (p=0.11) did not predict CSS. In contrast, T-stage (HR 1.24, 95% CI 1.05-1.48, p=0.014), the presence of metastases at the time of surgery (HR 3.91, 95% CI 1.90-8.04, p>0.001), tumour grade (HR 1.87, 95% CI 1.14-3.07, p=0.013), age in years (HR 1.06, 95% CI 1.03-1.09, p<0.001) and a CRP-value >5 mg/l (HR 2.67, 95% CI 1.28-5.54, p=0.009) were associated with poor CSS. Accordingly, the Kaplan-Meier 5-year CSS rates for patients with a CRP ≤ and > 5 mg/l were 54.2 and 26.4%, respectively (p=0.006, log rank; Figure 1).

Multivariate analysis including age and CRP as continuous metric parameters as well as sex, tumour stage, and differentiation identified the presence of lymph node and/or distant metastasis at the time of surgery (HR 4.24, 95% CI: 1.89-9.52; p<0.001, Cox regression), age [years] (HR 1.06, 95% CI 1.03-1.10, p<0.001), and the CRP-value [mg/l] (HR 1.01, 95% CI 1.004-1.02, p=0.001) as independent prognosticators for cancer-specific survival in patients with UUT-UC.

## Discussion

Only recently, Saito et al. [20] presented a study including 130 patients suggesting for the first time that elevated CRP is associated with a poor prognosis in patients with UUT-UC. The authors recommended stratifying patients according to the CRP cut-off values of 5.0 mg/l. In this study, we could confirm that a CRP value of >5.0 mg/l was significantly associated with muscle invasive disease (pT≥2), lymph node metastasis, distant metastasis and poor tumour differentiation. Furthermore, a high CRP value was associated with poor cancer specific survival in patients undergoing resection for UUT-UC. These results confirmed the impact of circulating CRP levels on the staging and prognosis of patients with UUT-UC.

Saito et al. [20] were able to identify an elevated CRP and lymph node invasion as an independent preoperative risk factor for disease-specific death. In this study multivariate analysis including age, sex, histological subtype, tumour stage, and differentiation identified the presence of lymph node and/or distant metastasis and both an elevated CRP value and age, at least as continuous metric parameters, as independent predictors for CSS in patients with UUT-UC.

As biomarkers in body fluids offer the opportunity for more objective and reproducible measurement prior to tumour surgery, the use of CRP as a worldwide well-standardized parameter, should not be underestimated. Rather than tumour tissue-based factors, it can easily be implemented as a prognostic factor in addition to tumour stage and grade, to more accurately stratify patients with UUT-UC. However, as CRP is a not a specific biomarker its use is limited in patients with other diseases in which CRP might be elevated, e.g. inflammatory or cardiovascular disease.

Systemic inflammatory response is of considerable importance in the relationship between the tumour, the host and outcome in patients with cancer. Elevation of the serum CRP level can be explained as part of a paraneoplastic syndrome [21]. It is likely that the association with muscle invasive disease among patients with high serum CRP levels may be due to tumor spread that cannot be detected either by routine imaging studies or by pathologic examinations.

However, the mechanism of high CRP in patients with solid tumors, e.g. urogential or gastrointestinal cancer, has not been determined yet. Several explanations have been proposed such as that CRP could directly impair immune functions [22-24] allowing unrestrained tumour growth. Alternatively, CRP is a component of the acute- phase response mainly induced by interleukin-6, and thus has the potential to enhance or inhibit the proliferation of carcinoma cells. Therefore, an elevated CRP might indicate tumours capable of producing significant amounts of proinflammatory cytokines, in particular interleukin-6 [25,26].

In genito-urinary malignancies, interleukin-6 functions as an autocrine growth factor for renal cell and prostate cancer [27-30]. Previously, we reported that a high preoperative serum CRP level is an independent predictor of poor survival in patients with renal cell cancer [17]. Trichopoulos et al. [18] revealed that elevated CRP can be related to a higher risk of developing bladder cancer. Experimental studies have shown that malignant urothelial cells acquire an additional growth advantage over benign cells by producing interleukin-6 [30]. These findings support the view that a systemic inflammatory response of the host indicates an aggressive nature of UUT-UC.

There are several limitations in our study that need to be acknowledged. The major limitation of this study is its retrospective design therefore the drawn conclusions have to be treated with reservation and need to be confirmed in prospective settings. Further limitations are the relative small number of patients and the short mean follow-up of only 28 month. In addition, surgical series have obvious inherent and selection biases as they only evaluate patients undergoing surgery and patient’s deemed nonsurgical candidates, for metastatic disease or other reasons, are not included. Finally, our cohort included significantly more patients with renal pelvis cancer than with UC of the ureter, this imbalance might have biased our analysis.

## Conclusions

This study confirms that a high preoperative CRP-level is significantly associated with muscle invasive and metastatic disease and that CRP can serve as an independent predictor of CSS. Its routine use could allow better risk stratification and risk-adjusted follow-up for patients with upper urinary tract urothelial carcinoma. However, our result should be validated in larger, multicenter studies.

## Competing interests

All authors declare that they have no competing interests.

## Authors’ contributions

BS carried out the data acquisition, participated in the data interpretation and drafting of the manuscript. AJS performed the statistical analysis and was involved in the drafting of the manuscript. GW carried out the data acquisition. CS and MAK revised the manuscript for important intellectual content. SS designed and supervised the study, participated in the data acquisition and interpretation as well as drafting of the manuscript. All authors read and approved the final manuscript.

## Pre-publication history

The pre-publication history for this paper can be accessed here:

http://www.biomedcentral.com/1471-2407/13/101/prepub
